# 2329. What Causes a Cluster to Grow? Factors Associated with a Nosocomial COVID-19 Cluster at a University Hospital in Central Tokyo

**DOI:** 10.1093/ofid/ofad500.1951

**Published:** 2023-11-27

**Authors:** Hilary Osaka, Yasuaki Tagashira, Yoshiaki Gu, Kunihiko Takahashi

**Affiliations:** Tokyo Medical and Dental University, Bunkyo-ku, Tokyo, Japan; Tokyo Medical and Dental University, Bunkyo-ku, Tokyo, Japan; Tokyo Medical and Dental University, Bunkyo-ku, Tokyo, Japan; Tokyo Medical and Dental University, Bunkyo-ku, Tokyo, Japan

## Abstract

**Background:**

Coronavirus disease 2019 (COVID-19) remains a significant threat to hospital infection control. Despite the decrease in severity and mortality associated with changes in the variants of concern and increased vaccination, SARS-CoV-2 transmissibility remains high, and nosocomial clusters continue to be reported in various countries. Although COVID-19 may be detected in multiple patients and healthcare workers (HCWs) in a given ward within a few days, it is uncertain whether these cases will develop into a large cluster.

**Methods:**

The present, retrospective cohort study of nosocomial COVID-19 clusters was conducted at Tokyo Medical and Dental University Hospital from January 2022 to January 2023 using data from the hospital’s infection prevention and control team. A nosocomial COVID-19 cluster was defined as consisting of more than 2 COVID-19 cases occurring within 5 days in the same ward. Cases with a clear epidemiological link and clusters outside the ward were excluded. We collected information regarding community trends, the index case details, and each cluster's ward profile. Predictors of cluster expansion to more than 5 cases were also examined.

**Results:**

66 clusters were identified at the hospital during the study period. The median number of COVID-19 cases per cluster was 3 (range: 2-22), and their median duration was 5 days (range: 1-27). 37 (56.1%), 25 (37.9%), and 4 (6.1%) clusters comprised both inpatients and HCWs, only HCWs, and only patients, respectively. The index case of 51 clusters (77.3%) was a HCW. 18 (27.3%) and 47 (72.7%) clusters contained > 5 and < 5 cases, respectively. No significant intergroup difference was found in terms of community prevalence, index case and ward characteristics, the rate of close contact or history of previous clusters. However, “clusters occurring during the non-epidemic period” and “infections > 3 occurring within 3 days” tended to develop into clusters containing more than 5 cases.

**Conclusion:**

Nosocomial clusters can occur anywhere, and predicting them is challenging. Further research is needed to identify the factors leading to cluster development and to establish effective infection prevention and control measures.

**Disclosures:**

**All Authors**: No reported disclosures

